# Use of Heated Tobacco Products within Indoor Spaces: Findings from the 2018 ITC Japan Survey

**DOI:** 10.3390/ijerph16234862

**Published:** 2019-12-03

**Authors:** Edward Sutanto, Danielle M. Smith, Connor Miller, Richard J. O’Connor, Andrew Hyland, Takahiro Tabuchi, Anne C. K. Quah, K. Michael Cummings, Steve Xu, Geoffrey T. Fong, Janine Ouimet, Itsuro Yoshimi, Yumiko Mochizuki, Maciej L. Goniewicz

**Affiliations:** 1 Division of Cancer Prevention and Population Sciences, Department of Health Behaviors, Roswell Park Comprehensive Cancer Center, Buffalo, NY 14263, USA; edward.sutanto@roswellpark.org (E.S.); Danielle.Smith@RoswellPark.org (D.M.S.); richard.o’Andrew.Hyland@RoswellPark.org (A.H.); 2 Cancer Control Center, Osaka International Cancer Institute, Osaka 537-8511, Japan; tabuchitak@gmail.com; 3 Department of Psychology, University of Waterloo, Waterloo, ON N2L 3G1, Canada; ackquah@uwaterloo.ca (A.C.K.Q.); s4xu@uwaterloo.ca (S.X.); geoffrey.fong@uwaterloo.ca (G.T.F.); j2ouimet@uwaterloo.ca (J.O.); 4 Department of Psychiatry & Behavioral Sciences, Medical University of South Carolina, Charleston, SC 29425, USA; cummingk@musc.edu; 5 Ontario Institute for Cancer Research, Toronto, ON M5G 0A3, Canada; 6 Division of Tobacco Policy Research, National Cancer Center Japan, Tokyo 104-0045, Japan; iyoshimi@ncc.go.jp; 7 Japan Cancer Society, Tokyo 100-0006, Japan; mochizuki@jcancer.jp

**Keywords:** heated tobacco products, heat-not-burn, secondhand exposure, involuntary exposure

## Abstract

Although heated tobacco products (HTPs) have become increasingly popular in Japan, little is known about whether these emerging tobacco products are being used within indoor public spaces. Nationally representative data were obtained prior to implementation of a comprehensive smoke-free law in Japan as part of Wave 1 of the International Tobacco Control Japan Survey (February–March 2018). We estimated the weighted prevalence of HTP use within indoor public spaces among tobacco users and compared these to estimates for combustible cigarettes (CCs). Overall, 15.6% of current tobacco users in Japan declared that they used HTPs within indoor public spaces. Any HTP use within indoor public spaces was significantly lower than any CC use (80.1% vs. 96.7%). Dual HTP + CC users reported using CCs more frequently than using HTPs within indoor public spaces (97.7% vs. 76.0%). In conclusion, HTP use is less common than CC use within indoor public spaces. Findings of this study can inform the development of targeted smoke-free policies to benefit public health.

## 1. Introduction

Since their introduction in 2014 [1], heated tobacco products (HTPs) have become increasingly popular in Japan. From 2015 to 2017, an estimated twelve-fold increase in past-30-day use of IQOS (a leading HTP brand) was observed among the Japanese general population, equating to millions of users nationwide [1]. As HTP use becomes more commonplace, it is important to examine potential health risks to non-users that may occur through secondhand exposure. While extensive research has implicated secondhand exposure to combustible cigarettes (CCs) as a cause of deleterious health effects [2,3], only a handful of independent studies have explored secondhand exposures to HTPs. These studies found that side-stream emissions from HTPs also contain many harmful constituents, and therefore may also present similar adverse health effects [4,5,6,7,8]. 

In an effort to protect bystanders from the harms of secondhand exposure, national smoke-free policies for indoor public spaces have been adopted by many countries [9]. In addition to improvement in health outcomes [10,11,12,13], the adoption of smoke-free policies has been tied to other public health benefits as well, such as lower consumption of CCs among smokers [14] and lower smoking initiation rates among youth [15]. Unlike other developed nations, Japan’s tobacco control policy has only recently incorporated a national smoke-free law in preparation for the upcoming 2020 Summer Olympic Games [16]. Upon enactment, this law will supposedly include restrictions on the use of HTPs within indoor public spaces. 

As Japan’s new national smoke-free law will be fully enforced on April 1, 2020 [16], there is a need to provide baseline estimates of tobacco product use within indoor public spaces before the law is enacted. In addition to describing the prevalence of indoor HTP and CC use in Japan, this study aimed to examine whether (1) the use of HTPs is more common within indoor public spaces than the use of CCs and (2) dual HTP and CC users use HTPs within indoor public spaces to circumvent smoke-free policy.

## 2. Materials and Methods 

### 2.1. Data Source and Analytic Sample

We analyzed data from the International Tobacco Control (ITC) Japan Survey Wave 1, a web-based survey conducted from February to March 2018. Participants were sampled from an existing panel that is nationally representative of Japanese adults, which includes both tobacco product users and non-users, aged 20 years and older (*n* = 4684). The main analytic sample in this study was current tobacco users (*n* = 4069), defined as participants who either use HTP or CC at least monthly. Exclusive users of HTPs (*n* = 170) or CCs (*n* = 3210) were defined as participants who only use one of either product, while dual users (*n* = 689) use both HTPs and CCs. Thus, any HTP users (*n* = 859) were defined as participants who currently use HTP at least once a month (exclusive HTP users and dual users), and any cigarette smokers (*n* = 3899) were participants who smoke CCs at least once a month (exclusive smokers and dual users). Ethical clearance (ORE#31428) was received from the Office of Research Ethics University of Waterloo.

### 2.2. Measures

HTP use within indoor public spaces was assessed using the following question: ‘In the last 30 days, have YOU used a heat-not-burn product in any of the following indoor public locations?’, and participants were instructed to respond for each of the following: (a) ‘At a restaurant or café’; (b) ‘At a bar or pub’; (c) ‘Indoors in your workplace’; (d) ‘In the public areas of multi-unit housing’; and (e) ‘On public transportation such as a bus, train, taxi, or in stations’. Participants were given the options ‘yes’, ‘no’, ‘refused’ (coded as missing), and ‘do not know’ (coded as missing). Participants who selected ‘yes’ to any of the five statements were classified as using HTP within any indoor public spaces, while participants who selected ‘no’ to all five statements were classified as not using HTP in any indoor public spaces. Responses to each of the statements were also reported separately in this study.

Participants’ (who selected ‘yes’ to the question, ‘In the last 6 months, have you visited a bar?’) use of CC in bars was assessed using the following question: ‘The last time you visited a bar, did you smoke indoors?’. CC use in restaurant or café was assessed similarly. Participants’ (who selected ‘yes’ to both following questions: (a) ‘Are you currently employed outside the home?’ and (b) ‘Do you usually work inside a building?’) use of CC in the workplace was assessed using the following question: ‘In the last 6 months, have you smoked within indoor areas where you work?’. Participants were given the options ‘yes’, ‘no’, ‘refused’ (coded as missing), and ‘do not know’ (coded as missing). Participants who selected ‘yes’ to any of the three specified settings were classified as using CCs within any indoor public spaces, while participants who selected ‘no’ to all three specified settings were classified as not using CCs in indoor public spaces. Unlike HTP, CC use was not evaluated in public areas of multi-unit housing and public transportation. 

Rules of smoking cigarettes in bars and pubs were examined using the following questions: ‘Which of the following best describe the rules about smoking cigarettes in bars and pubs where you live?’ Participants were given the options (a) ‘Smoking is not allowed in any indoor area’, (b) ‘Smoking is allowed only in designated/some indoor areas’, (c) ‘No rules or restrictions/Smoking is allowed in all indoor areas’, (d) ‘Refused’, or (e) ‘Do not know’. Rules about smoking cigarettes in the workplace was examined similarly, while for restaurant or café an additional option (‘Every restaurant, café has its own rules’) was given. 

### 2.3. Statistical Analysis

We presented the prevalence of HTP and CC use within indoor public spaces in weighted percentages and 95% confidence intervals (CI). Rao–Scott chi-square tests assessed the difference of use within indoor public spaces between HTPs and CCs. All statistical analyses were performed using ‘svy’ commands in Stata SE version 14.2 (StataCorp, College Station, TX, USA). Further details on the weighting strategy are provided in the ITC Japan Survey Technical Report (https://itcproject.org/files/JP1-1.5_Technical_Report_5June2019.pdf). We considered results statistically significant at *p* < 0.05 (two-tailed).

## 3. Results

**Figure 1:**
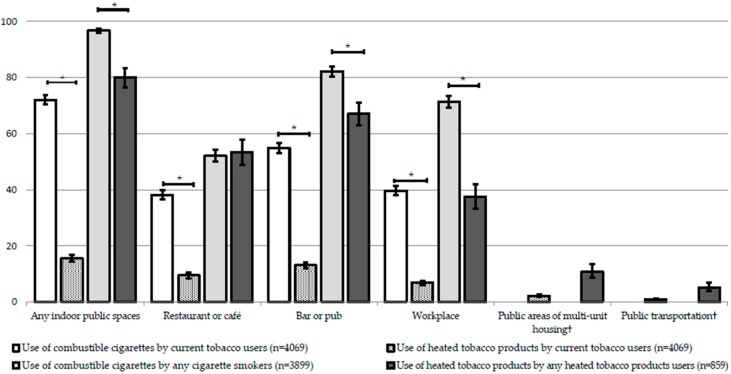
Use of tobacco product within indoor public places by current tobacco users, any cigarette smokers, and heated tobacco product users. The values represents weighted percentage. Error bars are 95% confidence intervals. * *p* < 0.001 (Rao-Scott Chi-Square tests) † The survey did not evaluate use of combustible cigarettes in public areas of multi-unit housing andpublic transportation.

Among current tobacco users, HTP use was significantly lower than CC use within any indoor public spaces (HTP: 15.6% [95%CI 14.4–16.8%]; CC: 72.0% [95%CI 70.4–73.7%], *p* < 0.001, Figure 1). Any HTP use within any indoor public spaces was significantly lower than any CC use (HTP: 80.1% [95%CI 76.5–83.3%]; CC: 96.7% [95%CI 95.9–97.4%], *p* < 0.001). While there was no significant difference inside restaurants or cafés, any HTP use was significantly lower than any CC use in bars or pubs (HTP: 67.1% [95%CI 63.0–71.1%]; CC: 82.3% [95%CI 80.5–84.0%], *p* < 0.001) and the workplace (HTP: 37.5% [95%CI 33.3–42.0%]; CC: 71.5% [95%CI 69.4–73.5%], *p* < 0.001). 

Table 1 presents comparisons of tobacco product use within indoor public spaces stratified by exclusive or dual product use. Dual users reported using CCs significantly more frequently than using HTPs within any indoor public spaces (CC: 97.7% [95%CI 95.7–98.7%]; HTP: 76.0% [95%CI 71.5–80.0%], *p* < 0.001). Within each of the three specified indoor public spaces (restaurant or café, bar or pub, and workplace), CCs were more frequently reported to be used by dual users rather than HTPs. Exclusive HTP users reported more frequent use of HTP within indoor public spaces than dual users (exclusive HTP users: 88.8% [95%CI 82.3–93.1%]; dual users: 76.0% [95%CI 71.5–80.0%], *p* = 0.002). Exclusive HTP users reported more frequent use of HTP than dual users in restaurants or cafés and bars or pubs; however, there was no significant difference in use within the workplace or on public transportation. 

There was a significant difference in the frequency of product use between any cigarette smokers and any HTP users (any cigarette smokers: daily use = 94.7% [95%CI 93.9–95.5%], weekly use = 4.6% [95%CI 3.9–5.3%], and monthly use = 0.7% [95%CI 0.4–1.0%]; any HTP users: daily use = 63.4% [95%CI 58.9–67.6%], weekly use = 16.1% [95%CI 13.5–19.2%], and monthly use = 20.5% [95%CI 16.7–24.9%], p < 0.001). There was no significant difference in the frequency of CC use between exclusive smokers and dual users (exclusive smokers: daily use = 94.8% [95%CI 93.9–95.5%], weekly use = 4.5% [95%CI 3.8–5.4%], monthly use = 0.7% [95%CI 0.4–1.0%], dual users: daily use = 94.4% [95%CI 91.9–96.2%], weekly use = 4.8% [95%CI 3.1–7.2%], monthly use = 0.8% [95%CI 0.3–2.2%]). There was a significant difference in the frequency of HTP use between exclusive HTP users and dual users (exclusive HTP users: daily use = 88.3% [95%CI 80.5–93.2%], weekly use = 9.9% [95%CI 5.7–16.7%], monthly use = 1.8% [95%CI 0.2–11.7%], dual users: daily use = 51.5% [95%CI 46.7–56.3%], weekly use = 19.1% [95%CI 16.1–22.6%], monthly use = 29.4% [95%CI 24.4–34.9%], *p* < 0.001). In bars and pubs, ‘smoking is allowed only in designated indoor areas’ (44.1% [95%CI 42.3–45.8%]) and ‘no rules and restrictions’ (33.8% [95%CI 32.2–35.5%]) were the most commonly reported cigarette smoking rules by current tobacco users. For restaurants or cafés, ‘smoking is allowed only in designated indoor areas’ (53.7% [95%CI 51.9–55.4%]) was the most common rule reported. In the workplace, ‘smoking is not allowed in any indoor area’ (51.3% [95%CI 49.0–53.5%]) and ‘smoking is allowed only in designated indoor areas’ (42.5% [95%CI 40.4–44.8%]) were the two most common cigarette smoking rules reported.

## 4. Discussion

To the best of our knowledge, this is the first study that collectively examined population estimates of HTP and CC use within indoor public spaces. In Japan, while the prevalence of HTP use within indoor public spaces is considerable, it is much lower compared to indoor use of CC. While differences in prevalence partly explain these findings (smoking remains much more common than HTP use) [17], there is still a difference between HTP and CC indoor public use even when it is stratified according to each subgroup of users. This may be due to at least three factors. First, this is likely a function of the previous lenient national law, which only stipulated that the property owner take necessary measures (without any penalty provisions) to protect against secondhand exposure [18]. Indeed, a substantial portion of participants in this study reported ‘smoking is allowed only in some indoor areas’ or ‘no rules or restrictions’ for cigarette smoking rules within indoor public spaces. Second, the observed difference could in part be explained by a higher frequency of product use for cigarettes smokers than for HTP users in this study. Thus, although both HTP users and cigarette smokers use their respective products at least monthly, cigarette smokers have more opportunities to be smoking indoors. Lastly, perceived smoking norms have been shown to influence individual behavior [19,20]. As opposed to an immediate trend, it is likely that smoking in indoor public spaces throughout Japan became a normalized practice gradually over time. The same could be true of HTPs as they continue to gain popularity. 

A previous study has noted the tobacco industry may use the claim that, since the tobacco is purportedly heated instead of burned, HTPs release no smoke and therefore can be excluded from indoor smoke-free policies [8]. At the time of survey, HTP use is allowed in smoke-free areas [21]. Of particular note is Japan Tobacco International’s Ploom TECH, which introduced a “No Smoking, Ploom TECH Only” concept to increase acceptance and normalize the use of Ploom TECH in indoor public spaces [22]. In principle, the new smoke-free law adopted in Japan will ban indoor smoking, however room designated for HTP use, akin to smoking room, in restaurants and bars is allowed as an exception [16]. It remains to be seen whether the new law will be enforced stringently enough to aid in lowering tobacco use within indoor public spaces. Future assessments studying the effect of the smoke-free law enactment on use of both CCs and HTPs within indoor public spaces will be needed to determine the impact of this new legislation. 

Dual users also reported using CCs more commonly than using HTPs within indoor public spaces. Studies that explored dual use patterns between CCs and alternative tobacco products, such as electronic nicotine delivery systems and HTPs, have reported that alternative tobacco products can serve as a complementary source of nicotine among certain subsets of cigarette smokers [23,24,25]. In a regulatory environment that imposed smoke-free law, dual users may switch from CCs to alternative tobacco products to circumvent the smoke-free environment. Such an explanation might not be applicable in the context of Japan as the regulatory environment for smoke-free policies at the time of survey was lacking. In Japan, many regulations for smoke-free workplaces are voluntarily implemented especially by large companies [26]. 

There are several limitations to this study. First, there was variation in the time frame used to assessed use of HTPs (last 30 days for all indoor public spaces) and CCs (last sixmonths for workplace and last time for bar and restaurant) within indoor public spaces. Second, although systematic reviews have shown smoke-free policies are associated with subsequent changes in smoking behavior [27,28], no causal relationship between the absence of a smoke-free law and tobacco product use within indoor public spaces can be made from these data, due to the cross-sectional nature of this study. Lastly, while frequency of product use was described in this study, we were unable to examine frequency of product use within indoor spaces.

## 5. Conclusions

In 2018, the use of CCs within indoor public spaces was more common than use of HTPs in Japan. Dual users reported using CCs more frequently than using HTPs within indoor public spaces. For public health benefits, smoke-free policy should be targeted toward both CCs and HTPs. Future studies evaluating the contribution of smoke-free laws in lowering tobacco product use (including HTPs) within indoor public spaces in Japan are warranted.

## Figures and Tables

**Table 1:** Comparison of tobacco product use in indoor public spaces stratified by exclusive or dual use.*

Location of Indoor Public Space	(A) HTPs by Exclusive HTP Users	(B) HTPs by Dual Users	(C) CCs by Dual Users	(D) CCs by Exclusive Smokers	Sig ^†^
	*n* = 170	*n* = 689		*n* = 3210	
	Weighted % (95% Confidence Interval)				
Any indoor public spaces	88.8 (82.3–93.1)	76.0 (71.5–80.0)	97.7 (95.7–98.7)	96.6 (95.7–97.3)	A-B: F(1, 842) = 10.07; *p* = 0.002
					B-C: F(1, 674) = 91.39; *p* < 0.001
					A-D: F(1, 2702) = 20.97; *p <* 0.001
Restaurant or café	61.1 (51.9–69.6)	49.7 (44.9–54.5)	61.5 (56.0–66.7)	51.3 (49.1–53.4)	A-B: F(1, 840) = 4.66; *p* = 0.031
					B-C: F(1, 672) = 17.75; *p* < 0.001
					A-D: F(1, 2605) = 4.19; *p* = 0.041
Bar or pub	74.2 (66.0–81.0)	63.7 (59.0–68.2)	89.2 (84.9–92.4)	81.5 (79.6–83.3)	A-B: F(1, 843) = 4.87; *p* = 0.027
					B-C: F(1, 674) = 73.29; *p* < 0.001
					A-D: F(1, 2412) = 4.23; *p* = 0.040
Workplace	39.6 (31.0–49.0)	36.5 (31.9–41.4)	76.0 (70.9–80.4)	71.0 (68.7–73.2)	A-B: F(1, 839) = 0.36; *p* = 0.547
					B-C: F(1, 672) = 151.72; *p* < 0.001
					A-D: F(1, 2049) = 47.80; *p* < 0.001
Public areas of multi-unit housing	6.0 (3.1–11.3)	13.1 (10.5–16.3)	NA ^‡^	NA ^‡^	A-B: F(1, 833) = 5.59; *p* = 0.018
Public transportation	3.5 (1.5–8.0)	6.2 (4.5–8.5)	NA ^‡^	NA ^‡^	A-B: F(1, 839) = 1.61; *p* = 0.205

Abbreviations: HTPs, heated tobacco products; CC, combustible cigarettes; NA, not applicable. * Exclusive users of HTPs or CCs were defined as participants who only use one of either products at least monthly, while dual users use both HTPs and CCs at least monthly. ^†^ Rao-Scott Chi-Square tests accounted the complex survey design. Resulting test stats were design-based F with respective degree of freedom for each pairwise comparison. ^‡^ The survey did not evaluate use of combustible cigarettes in public areas of multi-unit housing and public transportation.
